# Brittle-star mass occurrence on a Late Cretaceous methane seep from South Dakota, USA

**DOI:** 10.1038/s41598-018-27326-z

**Published:** 2018-06-25

**Authors:** Ben Thuy, Neil H. Landman, Neal L. Larson, Lea D. Numberger-Thuy

**Affiliations:** 1 Natural History Museum Luxembourg, Department of Palaeontology, 25, rue Münster, 2160 Luxembourg-city, Luxembourg; 2 American Museum of Natural History, Division of Paleontology (Invertebrates), New York, New York 10024 USA; 3 Larson Paleontology, Keystone, South Dakota, 57751 USA

## Abstract

Articulated brittle stars are rare fossils because the skeleton rapidly disintegrates after death and only fossilises intact under special conditions. Here, we describe an extraordinary mass occurrence of the ophiacanthid ophiuroid *Brezinacantha tolis* gen. et sp. nov., preserved as articulated skeletons from an upper Campanian (Late Cretaceous) methane seep of South Dakota. It is uniquely the first fossil case of a seep-associated ophiuroid. The articulated skeletons overlie centimeter-thick accumulations of dissociated skeletal parts, suggesting lifetime densities of approximately 1000 individuals per m^2^, persisting at that particular location for several generations. The ophiuroid skeletons on top of the occurrence were preserved intact most probably because of increased methane seepage, killing the individuals and inducing rapid cementation, rather than due to storm-induced burial or slumping. The mass occurrence described herein is an unambiguous case of an autochthonous, dense ophiuroid community that persisted at a particular spot for some time. Thus, it represents a true fossil equivalent of a recent ophiuroid dense bed, unlike other cases that were used in the past to substantiate the claim of a mid-Mesozoic predation-induced decline of ophiuroid dense beds.

## Introduction

Brittle stars, or ophiuroids, are among the most abundant and widespread components of the marine benthos, occurring at all depths and latitudes of the world oceans^1^. Most of the time, however, ophiuroids tend to live a cryptic life hidden under rocks, inside sponges, epizoic on corals or buried in the mud (e.g.^2^) to such a point that their real abundance is rarely appreciated at first sight.

As a result, openly visible mass occurrences of brittle stars raise attention and often incite scientific studies to explore the ecological factors explaining the alleged aberrant abundance (e.g.^3–8^). One of the most remarkable examples of a recent ophiuroid mass occurrence was discovered on the Macquarie Ridge chain of Seamounts in 2008 as part of the Census of Marine Life initiative: dubbed “Brittle star city”, the spectacular mass aggregation of ophiuroids was met with wide academic and public interest. That same year, the flanks of the Admiralty Seamount in Antarctica yielded a similarly impressive but smaller ophiuroid mass occurrence, consequently called “Brittle star village”^9^.

Interest in dense ophiuroid aggregations has gone far beyond the mere ecological background of the occurrences. Assumed fossil equivalents (e.g.^10–15^) have shaped the hypothesis that dense ophiuroid populations were common in shallow waters until the Jurassic and then significantly declined by the end of the Mesozoic (e.g.^16^). In combination with an assumed lower predation pressure in the Jurassic^16^, ophiuroid mass aggregations gained key witness status for the so-called Marine Mesozoic Revolution: a hypothesised causal connection between the evolution of durophagous predators and the Mesozoic decline of epifaunal suspension-feeder communities^17,18^. Although recent studies suggest that ophiuroid mass aggregations in modern oceans are neither rare nor restricted to low-predation areas (e.g.^8,19^), such communities are still commonly considered archaic benthic assemblages^20^.

We here describe an extraordinary fossil ophiuroid occurrence from the Late Cretaceous of South Dakota. The material is outstanding in several aspects. First, it represents one of the very few seep-associated ophiuroids known to date (e.g.^21^) and the first from the fossil record. Second, it is an unambiguous case of an autochthonous, dense ophiuroid community that persisted at a particular spot during several generations, thus representing a true fossil equivalent of a recent ophiuroid dense bed. As such, it casts doubt on several other cases of assumed fossil ophiuroid dense beds used in the past to substantiate the predation-induced decline of ophiuroid dense beds during the mid-Mesozoic (e.g.^22^). Finally, it significantly adds to the very sparse Mesozoic ophiuroid fossil record of the United States and the Western Interior Seaway in particular, currently counting as few as 9 species in total^23–29^. Apart from the systematic assessment of the material, we elucidate its intriguing taphonomy and discuss its significance for the interpretation of other fossil ophiuroid mass aggregations.

## Geological Context

Methane seep deposits have been recognized in the Upper Cretaceous marine sediments (shales) of the U.S. Western Interior since the late 1800 s and have been extensively studied over the last several decades^30–35^. The structures usually appear as conical mounds with their “core” or micritic bodies that were formed by the erosion of the surrounding, less consolidated sediments. These conical structures have been documented since the late 19th century and are frequently referred to as “tepee buttes”^36^.

Around the Black Hills, these deposits were formed from methane enriched fluids escaping from underlying layers of the Pierre, Niobrara, Belle Fourche, Mowery and Greenhorn Formations^35,37^. The presence of methane was confirmed through isotopic analyses of the carbonate cements^35,37–39^. Methane gas probably migrated along faults and fractures that developed in the Late Cretaceous prior to the uplift of the Black Hills during the Laramide Orogeny^34,35^. Dewatering of the thick, black shales (hundreds of meters thick) due to the weight of the water and sediments also, most likely, contributed to this process^35^.

Carbonate by-products precipitated out of solution as a result of anaerobic oxidation of methane thus allowing carbonate cementation to occur below the sediment water interface and in and around the fauna at the seeps that might have formed small, elevated mounds on the seafloor, creating refuges in an otherwise less than friendly environment^37,40,41^. Seeps may have represented semi-permanent self-sustaining habitats that were inhabited by a unique group of animals that lived on the nutrients provided by symbiotic, sulfur-oxidizing chemosynthetic bacteria^34,35,42^.

Echinoderms seem more common in the *Didymoceras cheyennense* and *Baculites compressus* zones around the Black Hills but are still mostly rare at seeps. Both regular and irregular echinoids have been found along with stalked crinoids^41^, feather stars (in preparation), asteroids^42^ and ophiuroids (this paper). An abundant food source and a hard substrate in an otherwise soft, muddy ocean bottom were most likely the key factors for echinoderms to habitat the seeps.

## Material and Methods

**Figure 1:**
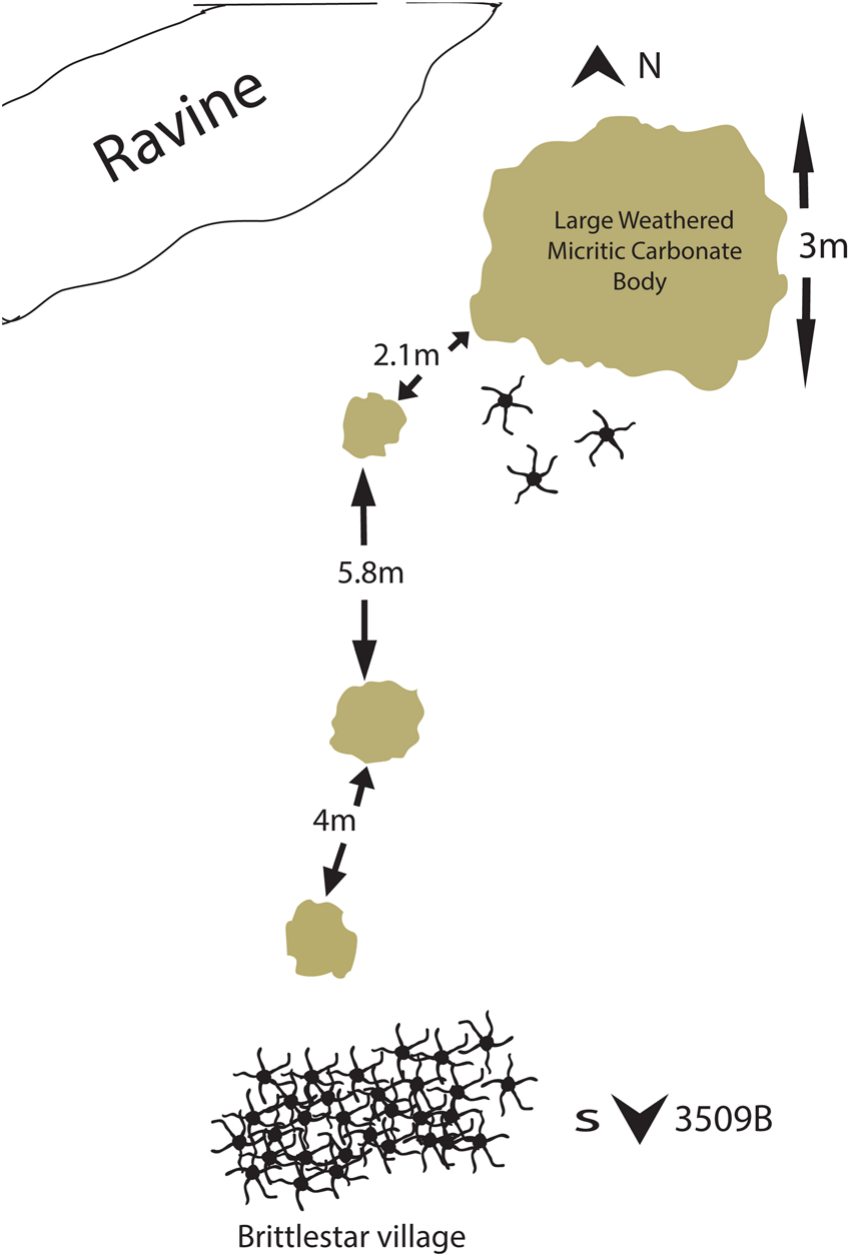
Site map of the ophiuroid-yielding seep site (AMNH locality 3509A) showing the location of the fossil ophiuroids, with three individuals near the large micritic body and a brittle-star mass aggregation (dubbed ‘brittle star village’) at some distance of it. The seep site is covered with seep associated carbonate concretions across the entire map area except for the ravine. Map courtesy of Jamie Brezina.

**Figure 2:**
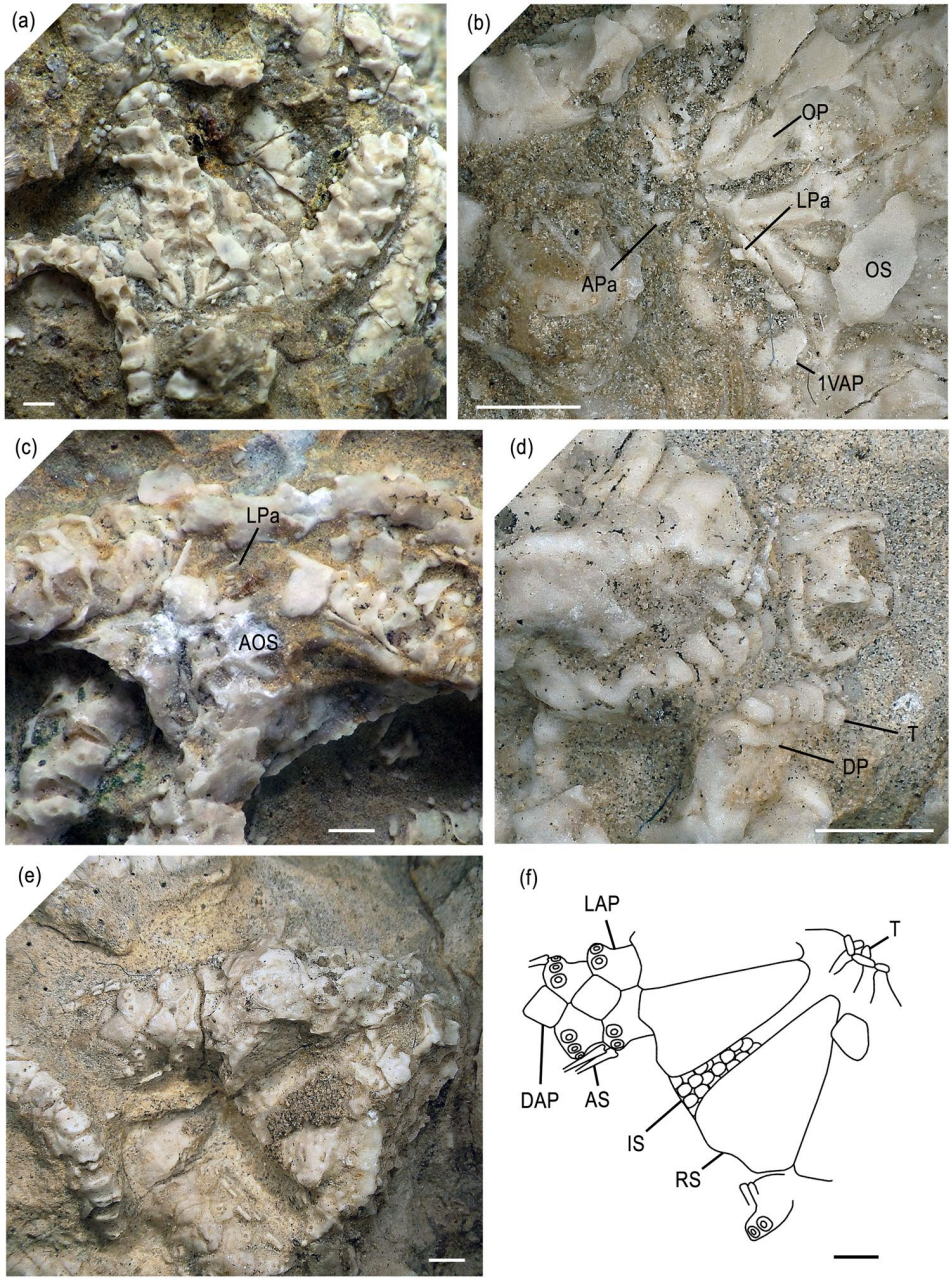
New ophiuroid *Brezinacantha tolis* gen. et sp. nov. from Upper Cretaceous (*Didymoceras cheyennense* Zone, upper Campanian) methane seep deposits of Pennington County, South Dakota. (**a**) AMNH 113563 (holotype), articulated skeleton exposing ventral side. (**b**) Detail of (**a**) showing mouth plating. (**c**) MnhnL OPH039 (paratype), articulated skeleton showing ventral side. (**d**) AMNH 113564 (paratype), detail of an articulated skeleton fragment exposing the dorsal side shown in (**e**), with dorso-lateral view of three dental plates with teeth. (**e**) AMNH 113564 (paratype), articulated skeleton fragment exposing the dorsal side. (**f**) interpretative drawing of the articulated skeleton fragment shown in (**f**). Abbreviations: AOS: adoral shield; APa: apical oral papilla; AS: arm spine; DAP: dorsal arm plate; DP: dental plate; IS: interradial disc scales; LAP: lateral arm plate; LPa: lateral oral papilla; OP: oral plate; OS: oral shield; RS: radial shield; T: tooth; 1VAP: first ventral arm plate. Scale bars equal 1 mm.

The material consists of 147 fragments of a carbonate plates yielding articulated ophiuroids, partially articulated arm fragments and fully dissociated skeletal plates. The ophiuroid locality is AMNH locality 3509A (WPT 130a) (Fig. 1), *Didymoceras cheyennense* Zone, upper Campanian, Late Cretaceous (Fig. 2), Pierre Shale, Pennington County, South Dakota. It is one of two closely associated seeps (WPT 130a & b) separated by approximately 25 m. Seep AMNH 3509A looks similar to many of the other highly weathered ‘teepee buttes’ while AMNH 3509B is almost indistinguishable. Ophiuroids have only been discovered from Seep AMNH 3509A.

Seep AMNH 3509A seems to have four different micritic masses. One is large and well exposed while the three others to the west are much smaller, meaning there was less methane seepage and thus less carbonate precipitation. There are no obvious carbonate structures exactly where the brittle stars were found on the seep (Fig. 1). While there are abundant seep associated carbonate concretions (SACS) all over the seep, the closest micritic mass is about 1.8 to 2 meters away. These carbonate concretions, burrows and pipes were probably precipitating just at or beneath the sediment/water interface.

AMNH 3509A has a rich, varied fauna that contains: ammonites (*Placenticeras meeki* (Böhm, 1898), *Hoploscaphites brevis* (Meek, 1876), *H. nodosus* (Owen, 1852), *Didymoceras cheyennense* (Meek and Hayden, 1856), *Spiroxybeloceras meekanum* (Whitfield, 1877) and *Baculites corrugatus* (Elias, 1933)), nautilus (*Eutrephoceras nebrascense* (Meek and Hayden, 1862)), ghost shrimp (*Callianassa* sp.), crabs (undescribed - Nyborg *et al*. in preparation), oysters, lucinids, inoceramids and other miscellaneous bivalves, gastropods, crinoids (*Lakotacrinus brezinai* Hunter *et al*.^41^), echinoids (regular), ophiuroid (this paper), tube worms, radiolarians, foraminifera, dinoflagellates, a mosasaur vertebra (*Plioplatecarpus*) along with a few miscellaneous fish vertebrae.

Selected ophiuroid skeletal plates were mechanically removed from one slab, mounted on a stub and gold-coated for Scanning Electron Microscopy using a Jeol Neoscope JMC-5000. Terminology follows Stöhr *et al*.^1^ and Thuy and Stöhr^43^. Ophiuroid classification is adopted from O’Hara *et al*.^44^. All types and additional specimens were deposited in the collections of the American Museum of Natural History (AMNH) and the Natural History Museum Luxembourg (MnhnL).

The electronic edition of this article conforms to the requirements of the amended International Code of Zoological Nomenclature, and hence the new names contained herein are available under that Code from the electronic edition of this article. This published work and the nomenclatural acts it contains have been registered in ZooBank, the online registration system for the ICZN. The ZooBank LSIDs (Life Science Identifiers) can be resolved and the associated information viewed through any standard web browser by appending the LSID to the prefix “http://zoobank.org/”. The LSID for this publication is: urn:lsid:zoobank.org:pub:3A98CFE8-7F81-4906-9D41-072CF02D61C5; for Brezinacantha: urn:lsid:zoobank.org:act:621CED39-46AF-4B98-B531-0CC362AAEE3E; for Brezinacantha tolis: urn:lsid:zoobank.org:act:BABFCFB5-F991-44EF-8DB6-A1A701187BCB. The electronic edition of this work was published in a journal with an ISSN, and has been archived and is available from the following digital repositories: PubMed Central, LOCKSS.

## Systematic Palaeontology

Class: Ophiuroidea Gray, 1840

Subclass: Myophiuroidea Matsumoto, 1915

Infraclass: Metophiurida Matsumoto, 1915

Superorder: Ophintegrida O’Hara *et al*., 2017

Order Ophiacanthida O’Hara *et al*., 2017

Suborder: Ophiacanthina O’Hara *et al*., 2017

Family: Ophiacanthidae Ljungman, 1867

Genus: *Brezinacantha* gen. nov.

Diagnosis: as for species

Type and only known species: *Brezinacantha tolis*™

Etymology: Genus named after Jamie Brezina (Rapid City, South Dakota) who discovered the ophiuroid specimens described herein, to honour his indefatigable commitment to the study of seep deposits and their fossil content in the Western Interior Seaway; suffix derived from ‘Acantha’, a Greek nymph whose name literally translates into “thorny”; gender feminine.

*Brezinacantha tolis* sp. nov.

Etymology: Species named after Sakis and Themis Tolis, founding members of a Greek Black Metal band, to honour their unorthodox yet powerful approach to music. The ophiuroids described herein merit in many ways a Black-Metal-referenced name: they gathered in great numbers in a spooky, toxic environment on top of their dead predecessors’ remains. The species name is entirely the responsibility of the lead author, Ben Thuy.

Holotype: AMNH 113563

Type locality and stratum: AMNH locality 3495 (WPT 130a), *Didymoceras cheyennense* Zone, upper Campanian, Late Cretaceous, Pierre Shale, Pennington County, South Dakota.

Diagnosis: Ophiacanthid with very large triangular radial shields accounting for three quarters of the disc radius, interradially separated by a narrow band of tiny, radially elongate scales; interradii not indented; two to three apical oral papillae; small, narrow adoral shields; distal portion of lateral arm plates strongly elevated, separated from remaining outer surface by a poorly defined, wavy ridge; up to five arm spines as long as one and a half arm segments, round in cross section, devoid of thorns.

Paratypes: AMNH 113564–113568, MnhnL OPH039 - 040

Other material examined: AMNH 113569–113585, AMNH 82689–82691, MnhnL OPH041 - 043.

Description of holotype: AMNH 113563 (Fig. 2a,b) is an articulated skeleton exposing the ventral side of three radii with associated proximal to median arm segments; reconstructed disc diameter 8.8 mm; ventral interradii straight to slightly concave, covered by few round scales, no granules or spines discernible; distal edges of radial shields visible, suggesting very large radial shields; oral shields as long as one quarter of the disc radius, rounded triangular, approximately as wide as long, with pointed proximal tip, slightly concave latero-proximal edges and convex distal edge; adoral shields narrow, inconspicuous; jaws moderately elongate and slender, with at least four small, conical, pointed lateral oral papillae in a single row and one or two similarly small, conical, pointed but shorter apical oral papillae; second oral tentacle pore opening within mouth slit.

Arms moderately wide, with first two segments covered by disc; first ventral arm plate wider than long, oval; following ventral arm plates nearly two times larger, nearly as wide as long, widest distally, with convex distal edge, deeply concave lateral edges and obtuse proximal angle; ventral arm plates separated by lateral arm plates from first arm segments onwards; no spurs or conspicuous ornamentation discernible. Lateral arm plates with conspicuously bulging distal portion sharply offset with respect to proximal portion of plate; elevated distal portion with at least four large, freestanding, ear-shaped spine articulations; stereom of lateral arm plate outer surface with slightly protruding trabecular intersections arranged in faint vertical rows; ventral portion of lateral arm plate not protruding ventro-proximalwards; spine articulations composed of narrow and strongly arched dorsal and ventral lobes proximally merged to form a circle around a large muscle opening, separated from much smaller nerve opening by sigmoidal fold; spine articulations increasing in size dorsalwards; large, conical, pointed arm spines at least as long as one arm segment. Tentacle openings small, covered by single oval tentacle scale.

Paratypes: MnhnL OPH039 (Fig. 2c) is a fragment of an articulated skeleton exposing the ventral side and preserving proximal to median portions of three arms; reconstructed disc diameter 8.7 mm; generally in agreement with holotype but with better preserved oral shields and lateral oral papillae; distal portion of oral shields with slighty concave latero-distal edges; at least four small, conical, pointed lateral oral papillae in a single row, proximalmost longest, almost spine-like.

AMNH 113564 (Fig. 2d–f) is a fragment of an articulated skeleton exposing the dorsal side and preserving a proximal arm portion; reconstructed disc diameter 10.9 mm; Interradius slightly concave but not indented; radial shields thick, isosceles triangular with slightly concave distal edges, accounting for three quarters of the disc radius, contiguous along the entire radial midline, separated interradially by numerous tiny, radially elongate scales; no scales or plates discernible in the centre of the disc; preservation too poor to ascertain the presence or absence of disc granules or spines. Centre of disc exposing non-fragmented, ventralwards widening dental plate with teeth preserved in place; five dorsalmost teeth pointed, dorsoventrally compressed, tetrahedral, in a single row with ventralward widening of the teeth; three ventralmost teeth smaller than dorsal ones, forming an irregular cluster. Arm segments higher than long; dorsal arm plates separated by lateral arm plates, lozenge-shaped with slightly obtuse distal and proximal edges and slightly pointed lateral edges, wider than long, devoid of conspicuous outer surface ornamentation; arm spines round in cross section, hollow, devoid of thorns or scale-like external ornamentation, as long as one and a half arm segments.

**Figure 3:**
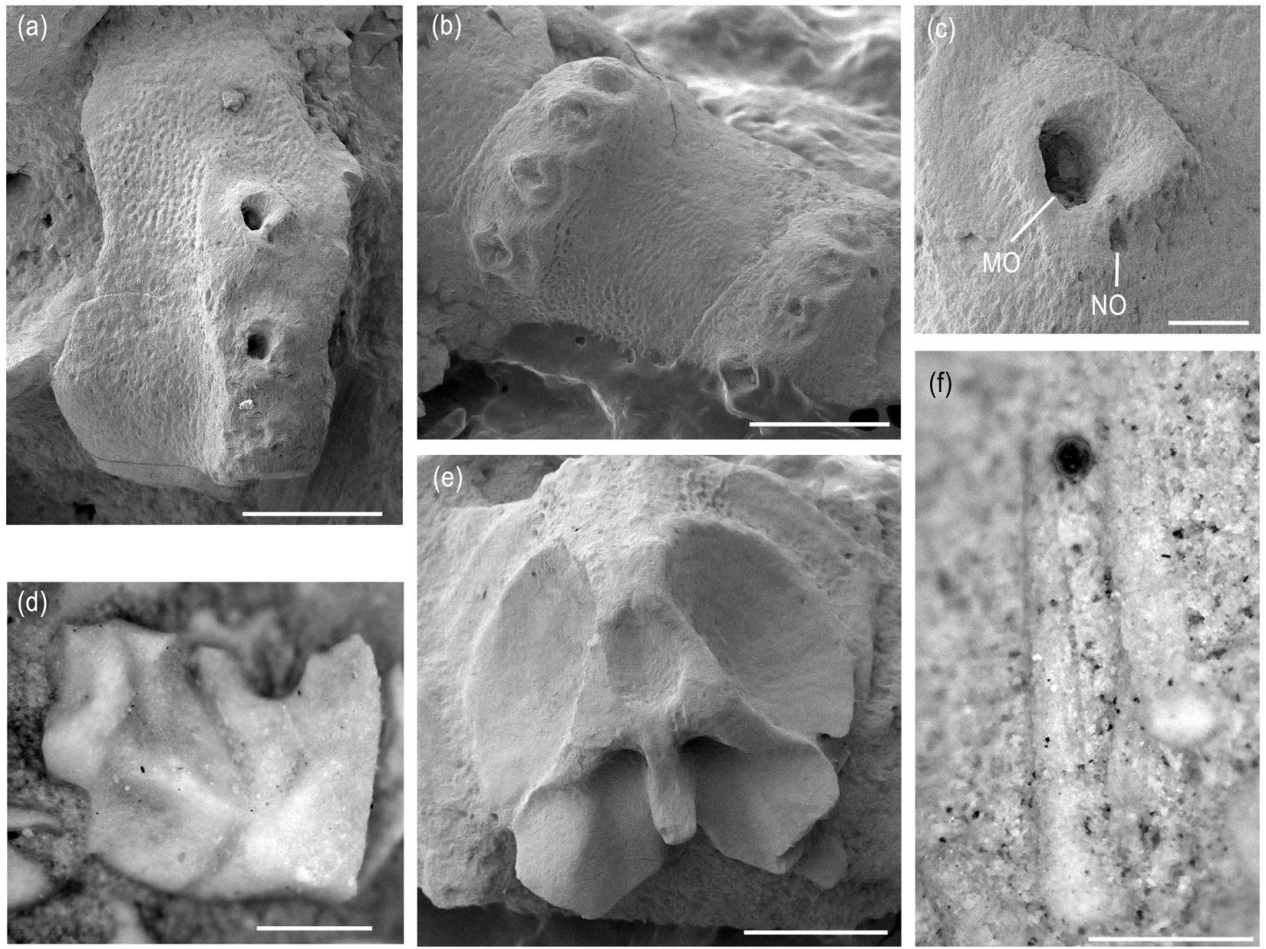
New ophiuroid *Brezinacantha tolis* gen. et sp. nov. from Upper Cretaceous (*Didymoceras cheyennense* Zone, upper Campanian) methane seep deposits of Pennington County, South Dakota. (**a**) MnhnL OPH040 (paratype), dissociated proximal lateral arm plate in external view, with dorsal side pointing upwards. (**b**) AMNH 113565 (paratype), median arm segments in lateral view, with dorsal side pointing downwards. (**c**) detail of (**a**) showing spine articulation. (**d**) AMNH 113566 (paratype), dissociated oral plate in adradial view. (**e**) AMNH 113567 (paratype), dissociated proximal vertebra in distal view, with dorsal side pointing upwards. (**f**) AMNH 113568 (paratype), dissociated arm spines, with proximal tip pointing downwards. Abbreviations: MO: muscle opening; NO: nerve opening. Scale bars equal 0.5 mm in (**a**,**b**) and (**d**–**f**), and 0.1 mm in (**c**).

MnhnL OPH040 (Fig. 3a,c) is a dissociated proximal lateral arm plate on a slab exposing the outer surface, approximately two times higher than long, with convex dorsal edge, wavy proximal edge and convex distal edge; ventral portion not protruding ventro-distalwards; poorly defined, small, horizontally elongate spur in the centre of the ventralmost third of the proximal edge; outer surface stereom with knobs slightly larger than surrounding pores and vertically elongate to form a faint, irregular striation especially in the central part of the outer surface; distal portion of lateral arm plate strongly elevated, dorsally fading into non-elevated dorsal portion of lateral arm plate, and proximally bordered by poorly defined, thin, wavy ridge; four large spine articulations freestanding on elevated distal portion of lateral arm plate; spine articulations with very large muscle opening encompassed by thin ventral and dorsal lobes proximally merged to form an almost perfect circle; much smaller nerve opening on ventro-distal edge of circle and separated from muscle opening by sigmoidal lobe; dorsalward increase in size of spine articulations, in size of gaps separating them and in the distance between the spine articulations and the distal plate edge.

AMNH 113565 (Fig. 3b) is a dissociated arm fragment composed of three median arm segments exposing the ventro-lateral side; lateral arm plate of middle arm segment well preserved, outer surface stereom as in MnhnL OPH040 but with slightly better developed vertical striation especially; elevated distal portion of the lateral arm plates extending over the entire height of the plate; four arm spine articulations similar to those of MnhnL OPH040.

AMNH 113566 (Fig. 3d) is a dissociated oral plate on a slab exposing the adradial face; oral plate slightly longer than high; adradial muscle attachment area small, in ventral position lining ventro-distal edge of articulation area.

AMNH 113567 (Fig. 3e) is a dissociated vertebra on a slab exposing the distal face, with strongly oblique, dorsalwards converging zygocondyles; zygosphene projecting beyond ventral edge of zygocondyles with projecting part almost as long as zygocondyles; dorso-distal muscular fossae not transformed into distalwards projecting process.

AMNH 113568 (Fig. 3f) are two dissociated arm spines, evenly tapering, conical, pointed, outer surface stereom coarse but devoid of thorns.

Remarks: The shape of the spine articulations and their dorsalward increase in size clearly place the present specimens in the ophiacanthid suborder Ophiacanthina. Within this group, the single row of lateral oral papillae, the absence of a ventral tooth cluster, the non-fragmented dental plate, the ornamentation of the outer lateral arm plate surface and the non-protruding ventral portion of the lateral arm plates suggest closest affinities with the family Ophiacanthidae. The excessively large radial shields in combination with the strongly elevated distal portion of the lateral arm plates and the relatively long arm spines place the present ophiuroids close to extant *Ophioplinthaca* Verrill, 1899. In this genus, however, the interradii are strongly indented and the radial shields are interradially bordered by a series of large, crescent-shaped scales (e.g.^45^).

Remarkable similarities are shared with the genus *Ophiosternle crinitum* (Quenstedt, 1876) from the Late Jurassic of Germany^46^, especially with respect to the size and position of the radial shields that are interradially separated by tiny scales. In the present specimens, however, the ventral tips of the jaws lack a well-defined cluster of apical papillae, the adoral shields are much narrower, the dorsal arm plates are smaller and not overlapping, and the arm spines are smaller and less numerous, thus precluding assignment to *Ophiosternle*. We thus suggest the new taxon *Brezinacantha tolis* to accommodate the ophiuroids described herein. With respect to the relevant morphological similarities, *Brezinacantha* seems the most closely related to *Ophioplinthaca* and *Ophiosternle*, and we anticipate close phylogenetic ties between the three.

## Taphonomy and Palaeoecology

The state of articulation of the ophiuroids mostly varies between the two extremes of the spectrum: fully dissociated skeletal plates on the one hand and fully articulated skeletons with no or little sign of post-mortem decay. The most likely mechanism to preserve an ophiuroid skeleton in its original anatomical disposition is rapid and effective burial either by storm-induced currents or by slumping (e.g.^47^). Yet, the site of discovery shows no sedimentary evidence for such an obrution event. Microbial and bacterial mats are a common feature of cold methane seeps and were highlighted as a possible factor in the preservation of partially articulated crinoids at neighboring seep locations^41^. Another very likely factor to explain the exceptional preservation of the ophiuroid skeletons is rapid carbonate cementation related to anaerobic oxidation of methane, known to enable exceptional fossil preservation in seeps (e.g.^48,49^). Considering the speed of post-mortem disarticulation of the ophiuroid skeleton, however, trapping and overgrowth by microbial mats and rapid cementation alone might not be sufficient to explain the here-described occurrence of fully articulated ophiuroid skeletons.

Interestingly, intact ophiuroid skeletons are known from sediments that lack obrution-related features but instead show signs of low oxygen levels at the time of deposition (e.g.^11,50^). These occurrences suggest that the otherwise rapid post-mortem decay of the ophiuroid skeleton can be significantly delayed by a lack of oxygen, an assumption corroborated by yet unpublished experiments on decay of dead ophiuroids exposed to high concentrations of methane or hydrogen sulfide (Y. Ishida, personal communication). We thus speculate that the ophiuroids described herein were killed by an increase in methane seepage, delaying skeletal decomposition and allowing microbial mats and rapid cementation to fossilise the skeletons intact.

**Figure 4:**
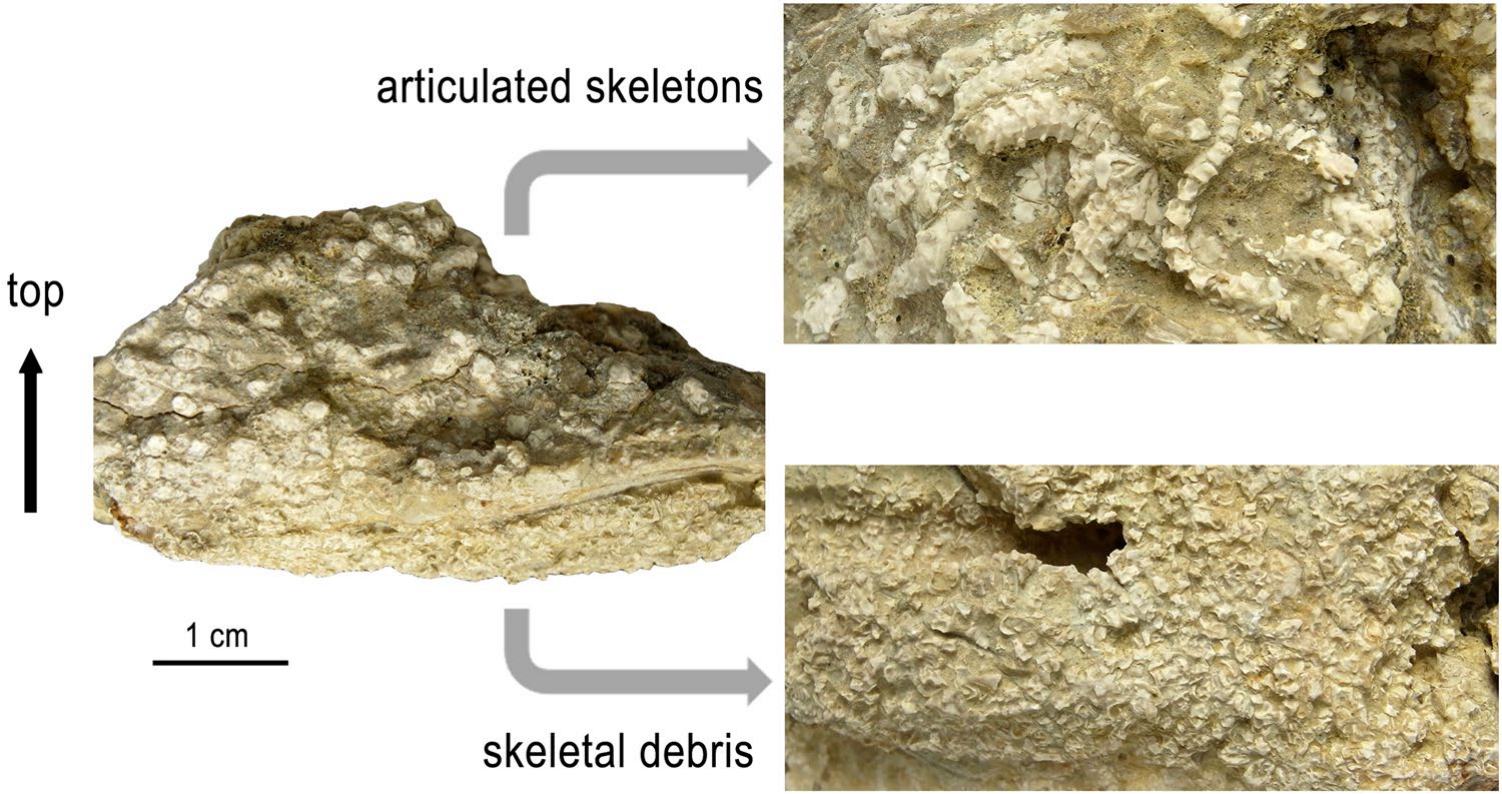
Slab with *Brezinacantha tolis* gen. et sp. nov. from Upper Cretaceous (*Didymoceras cheyennense* Zone, upper Campanian) methane seep deposits of Pennington County, South Dakota, AMNH 113563, in lateral view to show the densely packed articulated skeletons (top right detail: upside of slab) overlying an accumulation of dissociated skeletal plates (bottom right detail: underside of slab). Note the round arm sections in the lateral view.

With respect to the mode of occurrence, the limestone slabs yielding the ophiuroids all follow a striking pattern: the lower half of the slab is largely composed of an accumulation of dissociated skeletal plates, up to three centimeters thick and densely packed, often to an almost rock-forming extent, overlain, in the upper half of the slab, by densely packed, fully articulated skeletons, piled to layers up to 2.5 centimeters thick (Fig. 4). The dissociated skeletal plates mostly comprise vertebrae, lateral arm plates, oral plates and genital plates. Remarkably, arm spines are clearly underrepresented in most of the accumulations, in spite of being the most numerous component of the ophiuroid arm. Dissociated arm spines only occur in greater numbers underneath or inside bivalve and ammonite shell fragments. Long and hollow arm spines are among the first skeletal components of a disintegrated ophiuroid to be swept away by water movements (Thuy personal observation). We thus assume that the dissociated skeletal parts of the ophiuroid material described herein were size-sorted by weak bottom currents just strong enough to selectively wash away the arm spines except in protected spots, e.g. inside or underneath shell fragments.

Assuming the individuals preserved intact are a snapshot of the original mode of occurrence, the ophiuroids must have lived in a very dense aggregation with arms touching or even individuals overlapping, with an extrapolated density of approximately 1000 individuals per m^2^. Given the thickness of the ossicle accumulations underlying the articulated skeletons, and depending on the lifespan of the species, the dense ophiuroid aggregation must have persisted at that particular spot at least for several decades.

Judging from the distribution of the ophiuroid fossils at the site of discovery, the dense ophiuroid aggregation covered an area of at least 3 to 4 m, representing a fossil case of a brittle-star mass aggregation. It was found in an area with bivalve and ammonite shell hash and numerous seep-associated microbialite nodules at approximately 10 m distance from the central core of the seep (Fig. 1). Three small slabs with articulated remains of *Brezinacantha tolis* were found in the immediate vicinity of the central carbonate core, at some distance of the brittle-star mass aggregation. In analogy with modern relatives (e.g.^8^), *Brezinacantha* probably lifted its spinose arms in the water column to suspension feed, probably benefitting from a weak bottom water current (see above). The seep created a nutrient-rich environment with vast amounts of organic matter and thus provided abundant sources of food to sustain an extremely dense aggregation of suspension-feeding ophiuroids for several generations.

## Dense Ophiuroid Aggregations in the Fossil Record

Proponents of the Marine Mesozoic Revolution postulate that dominance within communities of suspension-feeding invertebrates was pushed to more cryptic, active, or heavily armoured species by increased predation pressure in the course of the Mesozoic (e.g.^17,18^). In the same logic, a Mesozoic decline of brittle-star beds visible in the fossil record was postulated, in association with the diversification of durophagous predators^22^, except in areas with anomalously low predation (e.g.^5^), e.g. at high latitudes (e.g.^15^).

In an attempt to understand the ophiuroid mass occurrence described herein in a broader evolutionary context, we noticed that the compilation of fossil ophiuroid beds used to substantiate the assumed mid-Mesozoic decline of such occurrences^22^ vary considerably in their taphonomic and palaeo-ecological context. Critical re-evaluation of the occurrences compiled by Aronson^22^, including direct examination of the specimens in several cases, suggests that some might, in fact, not represent fossil equivalents of recent shallow-water ophiuroid beds. Some consist of a series of articulated ophiuroids collected at the same locality but probably not forming a dense bed originally (Early Cretaceous of Alexander Island, Mid-Jurassic of Weymouth, Late Devonian of Velbert and Angerbachtal). Others are the result of storm-induced obrution, implying that the ophiuroids might not be autochthonous but instead washed together before burial (Early and Mid-Triassic records). Finally, the Mid-Jurassic occurrence from La-Voulte-sur-Rhône, while probably representing a true fossil ophiuroid mass occurrence, originates from a bathyal rather than shallow-water setting^51^. Furthermore, from a methodological point of view, it should be noted that the Late Devonian records of Velbert and Angerbachtal and the Mid-Triassic records of Nordwürttemberg, Weimar and Mergentheim originate from the same depositional settings respectively, implying that they should possibly be considered as a single Late Devonian and a single Mid-Triassic occurrence.

With so many dubious, if not invalid, fossil occurrences in the original compilation, and with the number of true fossil ophiuroid dense beds described since including the present one, the hypothesis of the mid-Mesozoic decline of ophiuroid dense beds should be revisited.
